# A Landscape of Metabonomics for Intermingled Phlegm and Blood Stasis and Its Concurrent Syndromes in Stable Angina Pectoris of Coronary Heart Disease

**DOI:** 10.3389/fcvm.2022.871142

**Published:** 2022-05-13

**Authors:** Li Zheng, Zhang Mingxue, Li Zeng, Zhou Yushi, Ao Yuhan, Yang Yi, Liu Botong

**Affiliations:** First Clinical College, Liaoning University of Traditional Chinese Medicine, Shenyang, China

**Keywords:** metabonomics, intermingled phlegm and blood stasis, Qi stagnation, Qi deficiency, stable angina pectoris of coronary heart disease, toxin

## Abstract

**Objectives:**

In this study, we analyzed the metabonomics of intermingled phlegm and blood stasis (IPBS) and its three concurrent syndromes in patients with stable angina pectoris of coronary heart disease.

**Methods:**

A total of 164 sera of separated outpatients from 12 national tradition Chinese medicine clinical research centers with IPBS or concurrent syndromes were collected for the study and assessed with LC-ESI-MS/MS (liquid chromatography—electrospray ionization tandem—mass spectrometry)-based metabolomics and multivariate statistical analysis.

**Results:**

Non-differential metabolites between IPBS and its separate syndrome combined with the top 100 most abundant metabolites in four groups were screened to reflect the essence of IPBS. Amino acid and its metabolomics and glycerol phospholipids were screened for common metabolites, and these metabolites were mainly enriched in valine, leucine, and isoleucine metabolism and glycerophospholipid metabolism. Principal component analysis revealed that the difference between IPBS and its separate concurrent syndromes was not distinct. Compared with IPBS, anserine, cytidine 5′-diphosphocholine, and 7,8-dihydro-L-biopterin separately significant increase in phlegm stasis and toxin (PST), phlegm stasis and Qi stagnation (PQS), and phlegm stasis and Qi deficiency (PQD). While these different metabolites were associated with histidine metabolism, beta-alanine metabolism, glycerophospholipid metabolism, and folate biosynthesis. Three accurate identification models were obtained to identify the difference between IPBS and its concurrent syndromes.

**Conclusion:**

Our study indicated that valine, leucine, and isoleucine metabolism and glycerophospholipid metabolism could represent the essence of IPBS; dysregulated metabolites were valuable in identifying PST from IPBS.

## Introduction

Coronary heart disease is a kind of disease that seriously endangers public health. In China, the number of patients with coronary heart disease is increasing year by year, reaching up to 11 million (1). And death caused by cardiovascular disease has accounted for more than 40% of resident deaths due to disease, ranking first, higher than tumors and other diseases (2). Thus, more attention is paid to coronary heart disease. Stable angina is one of the main types of coronary heart disease, with a high incidence rate, frequent onset, long course, and healing resistance (3). If not treated in time, it can easily cause heart failure and death. Clinical practice has proved that traditional Chinese medicine (TCM) has unique advantages in the treatment of coronary heart disease (4, 5). However, accurate syndrome differentiation is indeterminate, which affects the basis for the effective role of TCM (6). TCM mostly establishes the syndrome type by “observing inside function through outside change.” The external performance of typical symptoms, tongue, and pulse are examined to reflect the inside disorders of patients (7). However, this diagnostic method has obvious limitations. The state of patients and the experience of doctors are interference factors for syndrome differentiation. Recently, new technologies have been applied to TCM syndrome differentiation to form a new direction for accurate identification (8, 9). Metabonomics is such a technology to reveal the role of diseases and drugs *in vivo* through quantitative analysis of metabolites (10). It can be used for explaining the essence of TCM types and accurate syndrome differentiation, providing an objective and accurate basis for the diagnosis and treatment of diseases.

In TCM, angina pectoris of coronary heart disease mostly belongs to the syndrome of deficiency, excess, or the mixture of deficiency and excess (11). The mainstream theory suggest that deficiency refers to Qi deficiency, Qi and Yin deficiency, or Yang deficiency in the heart. The essence of deficiency is phlegm turbidity, water drinking, and blood stasis (11). Research showed that intermingled phlegm and blood stasis syndrome (IPBS) mainly related to phlegm turbidity and blood stasis, indicating that syndrome differentiation of IPBS was valuable for clinical diagnosis of coronary heart disease. However, IPBS mostly exists as the main syndrome, therefore it also coexists with other symptoms, such as Qi deficiency, Qi stagnation, or toxins. These symptoms are concurrent and cause confusion for accurate syndrome differentiation. Thus, accurately distinguishing simple main symptoms and mixed concurrent symptoms and obtaining a proper selection for treatment are particularly important. Therefore, we conducted a multicenter and refined clinical study, using wide-target metabonomics technology to screen the major metabolites and differentially expressed metabolites to reflect the essence of IPBS and accurately distinguish IPBS from its concurrent syndromes.

## Materials and Methods

### Patients

Patients in this study were from 12 national TCM clinical research centers between June 2019 to August 2020. The 12 national centers included Affiliated Hospital of Liaoning University of traditional Chinese medicine, Dongzhimen Hospital of Beijing University of traditional Chinese medicine, Affiliated Hospital of Shaanxi University of traditional Chinese medicine, the First Affiliated Hospital of Guangxi University of traditional Chinese medicine, Yunnan hospital of traditional Chinese medicine, Affiliated Hospital of Southwest Medical University, Longhua Hospital Affiliated to Shanghai University of traditional Chinese medicine, the First Affiliated Hospital of Guangzhou University of traditional Chinese medicine, Hubei Hospital of traditional Chinese medicine, Gansu Central Hospital, the Affiliated Hospital of Chengdu University of traditional Chinese medicine, and the First Affiliated Hospital of Heilongjiang University of traditional Chinese medicine. The Ethics Committee of each research center approved and supervised the research protocol. Patients strictly met the diagnostic of IPBS or related concurrent syndromes in stable angina of coronary heart disease.

These patients ranged from 35 to 75 years old. They had not taken any traditional Chinese medicine within 2 weeks. All patients were voluntarily participating in clinical observation, cooperated with follow-up, and signed informed consent. The included patients were asked to avoid eating high-fat or high-protein foods, drinking alcohol for 1 day, and fast for 8–12 h before blood collection. Patients with any of the following conditions were excluded from the research.

(a) Heart failure with valvular heart disease, various types of cardiomyopathy, malignant arrhythmia, and NYHA cardiac function grades III–IV.
(b) Patients with untreated or uncontrolled hypertension (BP ≥ 180/110 mmHg).
(c) Patients with severe cerebrovascular disease.
(d) Patients with severe pulmonary insufficiency (PaO2 < 60 mmHg), serious primary diseases of the endocrine and hematopoietic system, moderate and severe liver insufficiency (aminotransferase level is three times higher than the upper limit of the normal value), moderate and severe renal insufficiency (eGFR < 60 ml/min/1.73 m2), or new acute cerebrovascular diseases in the last 3 months.
(e) Psychiatric patients.
(f) Pregnant or lactating patients.
(g) Patients who participated in other clinical trials in the last 3 months.
h. Patients with life expectancy shorter than 1 year.
(i) Patients with an allergic constitution, or those allergic to traditional Chinese medicine;
(j) Patients that were unwilling or unable to accept clinical follow-up.

### Diagnosis Criteria

The diagnosis of IPBS or related concurrent syndromes in stable angina of coronary heart disease strictly referred to the national standards, industrial standards, expert consensus, and other high-level evidence to formulate the inclusion criteria, curative effect indicators, clinical standard operation steps, and other content. The diagnostic criteria of Western medicine refer to the guidelines for the diagnosis and treatment of chronic and stereotyped angina pectoris, while the diagnostic criteria of TCM refer to guiding principles for clinical research of new traditional Chinese medicine, TCM clinical diagnosis and treatment terminology and syndrome (national standard, GB/phylum 16751.2-1997), TCM syndrome differentiation criteria of coronary heart disease, research on macro diagnostic criteria of intermingled phlegm and blood stasis of coronary heart disease, and the diagnostic criteria of curative effect of TCM diseases and syndromes. Combined with expert demonstration, we formulated the TCM diagnostic criteria of IPBS and refined the concurrent syndrome elements of IPBS of stable angina pectoris in coronary heart disease. The concurrent syndrome was judged by two professional chief physicians of TCM. If there was any disagreement, the decision was reviewed and determined by another professional chief physician of TCM to ensure the accuracy of syndrome differentiation.

The diagnostic criteria for stable angina of coronary heart disease were formulated in combination with the 2007 Chinese guidelines for the diagnosis and treatment of chronic stable angina pectoris, the 2013 ESC guidelines for the management of stable coronary heart disease, the update of the 2014 guidelines for the diagnosis and management of patients with stable ischemic heart disease jointly issued by ACC/AHA/AATS/PCNA/SCAI/STS, and the expert consensus on the non-invasive imaging path of stable coronary heart disease in 2017. Stable angina of coronary heart disease included symptoms of precordial discomfort and any of the following conditions.

(a) Definite ST-T dynamic changes in ECG during the onset of chest pain, or ST segment depression or T wave inversion in resting ECG, but it showed “pseudo normalization” during the onset of chest pain; or 24-h ambulatory ECG showed ST-T changes consistent with symptoms.
(b) For those with abnormal resting ECG, LBBB, ST segment decline > 1 mm, pacing rhythm, preexcitation syndrome, and other ECG exercise tests that are difficult to accurately evaluate, and a positive load function test (echocardiography, radionuclide myocardial perfusion imaging, cardiac magnetic resonance imaging).
(c) Bruce protocol was used, and the exercise test was positive.
(d) Coronary CT or coronary angiography showed at least one main branch or main branch stenosis > 50%.

Diagnostic criteria for IPBS were formulated with reference to the 2008 guidelines for the diagnosis and treatment of common diseases in internal medicine of TCM (diseases of Western Medicine), syndrome of terms in clinical diagnosis and treatment of TCM, guiding principles for clinical research of new traditional Chinese medicine (2002 Edition), and diagnostic efficacy standards of diseases and syndromes of TCM (2012 Edition) in combination with expert demonstration. Diagnosis of IPBS included the main symptoms below, two or more secondary symptoms, combined with tongue and pulse signs.

(a) Main symptoms: chest tightness, chest (or precordial) pain, stinging pain, or stuffy (or severe) pain.
(b) Secondary symptoms: heavy head and body sleepiness, nausea, vomiting or generalized vomiting, weight increase, excessive phlegm, shortness of breath, and a cyan (or light purple or dark purple) complexion of the lips, nails, or skin.
(c) Tongue and pulse: purple tongue (light purple or dark purple or cyan), ecchymosis, greasy or slippery fur, and astringent or slippery pulse string.

On the basis of symptoms of IPBS, diagnosis of concurrent syndromes also had the following conditions. Phlegm stasis and Qi stagnation (PQS) included chest, back, and flank rib pain, epigastric stuffiness, and pulse string. Phlegm stasis and Qi deficiency (PQD) included recurrent chest pain, chest tightness, shortness of breath, wheezing when moving, palpitation, easy to sweat, tiredness and lazy speech, pale complexion, dark tongue or tooth marks, thin white fur, weak pulse, or generation. Phlegm stasis and toxin (PST) includes shortness of breath with wheezing, or with red spots on the face or brocade patterns, or body rash, purple (or dark), crimson tongue, dark haggard tongue, powder moss, and pricked (red, white, or black) tongue or swollen tongue.

### Sample Preparation and Extraction

A total of 4 ml of venous blood was collected from each enrolled patient, then centrifuged at 3,500 r/min for 10 min. The serum was taken and frozen at -80°C. Then, the sample was thawed on ice, vortexed for 10 s and mixed well, 300 μl of pure methanol was added to 50 ul of plasma/serum, mixed for 3 min, and centrifuged at 12,000 rpm at 4°C for 10 min. Then we collected the supernatant and centrifuged it at 12,000 rpm at 4°C for 5 min. It was left in a refrigerator at -20°C for 30 min, centrifuged at 12,000 r/min at 4°C for 3 min, and 150 ul of the supernatant was added to the liner of the corresponding injection bottle for on-board analysis.

### Metabolic Detection Process

Overall, 164 sera sample extracts were analyzed using an LC-ESI-MS/MS system (UPLC, ExionLC AD, MS, QTRAP^®^ System).^1,2^ The T3 UPLC analytical conditions were as follows: UPLC column, Waters ACQUITY UPLC HSS T3 C18 (1.8 μm, 2.1 mm*100 mm); column temperature, 40°C; flow rate, 0.4 mL/min; injection volume, 2 μL; solvent system, water (0.1% formic acid): acetonitrile (0.1% formic acid); and gradient program, 95:5 V/V at 0 min, 10:90 V/V at 11.0 min, 10:90 V/V at 12.0 min, 95:5 V/V at 12.1 min, and 95:5 V/V at 14.0 min. The Amide UPLC analytical conditions were as follows: UPLC column, Waters ACQUITY UPLC BEH Amide (1.7 μm, 2.1 mm*100 mm); column temperature, 40°C; flow rate, 0.4 mL/min; injection volume, 2 μL; solvent system, water (20 mM ammonium formate and 0.4% ammonia): acetonitrile; and gradient program, 10:90 V/V at 0 min, 40:60 V/V at 9.0 min, 60:40 V/V at 10.0 min, 60:40 V/V at 11.0 min, 10:90 V/V at 11.1 min, and 10:90 V/V at 15.0 min. LIT and triple quadrupole (QQQ) scans were acquired on a triple quadrupole-linear ion trap mass spectrometer (QTRAP) QTRAP^®^ LC-MS/MS System, equipped with an ESI Turbo Ion-Spray interface, operating in positive and negative ion mode and controlled by Analyst 1.6.3 software (Sciex). The ESI source operation parameters were as follows: source temperature, 500°C; ion spray voltage (IS), 5,500 V (positive), -4,500 V (negative); ion source, gas I (GSI), gas II (GSII), curtain gas (CUR) was set at 55, 60, and 25.0 psi, respectively; the collision gas (CAD) was high. Instrument tuning and mass calibration were performed with 10 and 100 μmol/l polypropylene glycol solutions in QQQ and LIT modes, respectively. A specific set of MRM transitions was monitored for each period according to the metabolites eluted within this period.

### Analysis Process

After obtaining the spectrum data of metabolic substances of different samples, the characteristic ions of each substance were screened by triple quadrupole scans, and the mass spectrometry was quantitatively analyzed by Analyst 1.6.3 and MultiQuant 3.0.3 software. The data were uploaded in MetaboLights.^3^ The multivariate statistical analysis method was used to construct a reliable mathematical model. Unsupervised PCA (principal component analysis) was performed by R.^4^ The data were unit variance-scaled before unsupervised PCA. Significantly regulated metabolites between groups were determined by VIP > 1 and absolute log2 (fold change) > 1. VIP values were extracted from the OPLS-DA result, which also contained score plots and permutation plots, and generated using R package MetaboAnalystR. The data were log-transformed (log2) and mean-centered before OPLS-DA. Identified metabolites were annotated using the KEGG compound database.^5^ Annotated metabolites were then mapped to KEGG pathways through online software MetaboAnalyst5.0.^6^ Significantly enriched pathways were identified with a hypergeometric test’s *P*-value.

## Results

### Characteristics of Included Samples

In the study, there were 164 sera from separated outpatients with IPBS or concurrent syndromes, including 64 cases of IPBS, 64 cases of phlegm stasis and Qi deficiency (PQD), 26 cases of phlegm stasis and Qi stagnation (PQS), and 10 cases of phlegm stasis and toxin (PST). There was no difference in gender, age, weight, fasting blood glucose, triglyceride (TG), total cholesterol (TC), low density lipoprotein C (LDL-C), and history of hypertension and diabetes among the four group of samples (Table 1), indicating no population difference in the experiment.

**TABLE 1:** Characteristics of included samples.

Syndrome		Intermingled phlegm and blood stasis	Phlegm stasis and Qi deficiency	Phlegm stasis and Qi stagnation	Phlegm stasis and toxin	*P*-value
Sample		64	64	26	10	
Age (year)		62.11 ± 7.32	61.27 ± 8.26	59.31 ± 9.19	59.60 ± 9.61	0.06
Weight (kg)		66.66 ± 9.76	70.84 ± 13.76	72.86 ± 10.18	67.55 ± 8.54	0.53
Gender	Male	48	36	23	8	0.74
	Female	16	28	3	2	
Hypertension	Yes	43	48	17	5	0.75
	No	21	16	9	5	
Diabetes	Yes	18	16	7	3	0.49
	No	46	48	19	7	
Fasting blood glucose (mmol/L)		6.20 ± 1.92	6.29 ± 1.72	6.38 ± 2.04	6.39 ± 1.55	0.14
TG (mmol/L)		1.51 ± 0.76	1.78 ± 0.99	1.77 ± 1.09	1.74 ± 0.89	0.92
TC (mmol/L)		4.03 ± 1.130	4.19 ± 1.03	3.91 ± 0.94	3.91 ± 2.03	0.38
LDL-C (mmol/L)		2.23 ± 0.97	2.41 ± 0.87	2.25 ± 0.81	2.43 ± 2.12	0.78

### Characteristics of Metabolic Results

**FIGURE 1:**
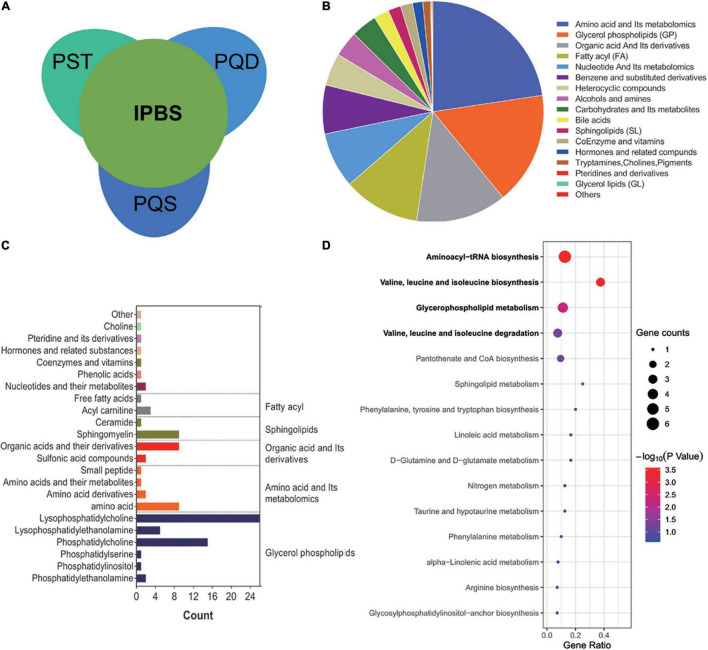
Characteristics of metabonomics. **(A)** Interrelation of IPBS and its related concurrent syndromes, PST, PQD, and PQS. IPBS, intermingled phlegm and blood stasis; PST, phlegm stasis and toxin; PQD, phlegm stasis and Qi deficiency; PQS, phlegm stasis and Qi stagnation. **(B)** The pie chart shows the main components of 800 metabolites in the primary class. Each color represents a separate metabolite. **(C)** The main secondary structure of intersection among top 100 metabolites in each group. A total of 96 metabolites are included and glycerol phospholipids are the main components. **(D)** KEGG analysis of 96 metabolites. The gene ratio represents the enrichment factor, the bubble scale represents the number of different genes, the depth of the bubble color represents the *P*-value.

Based on the interrelation of IPBS and its related concurrent syndromes, a diagram was drawn to reflect the overall inclusion and divergence relationships. The major symptom of IPBS had an intersection with the related concurrent syndromes PQS, PQD, and PST. Also, the concurrent syndromes were separately different to the major symptom of IPBS (Figure 1A). In this study, 800 metabolites, including 17 primary class and 43 secondary class metabolites, were detected. In the primary class level, amino acids and their metabolites, glycerol phospholipids, organic acid and its derivatives, fatty acyl, and benzene and its derivatives were major components, with a cumulative proportion of 78.88% (Figure 1B). To reflect an overview of the metabolome, the top 100 most abundant metabolites in each group were screened, and an intersection of the top expressed metabolites in each group was obtained. A total of 96 metabolites with 12 primary classes were selected; glycerol phospholipids were the main components (Figure 1C). Then these 96 metabolites were enriched in KEGG pathway analysis. The significantly enriched pathways were aminoacyl-tRNA biosynthesis; valine, leucine, and isoleucine metabolism; and glycerophospholipid metabolism (Figure 1D).

### Comprehensive Analysis to Reveal the Essence of Intermingled Phlegm and Blood Stasis

**FIGURE 2:**
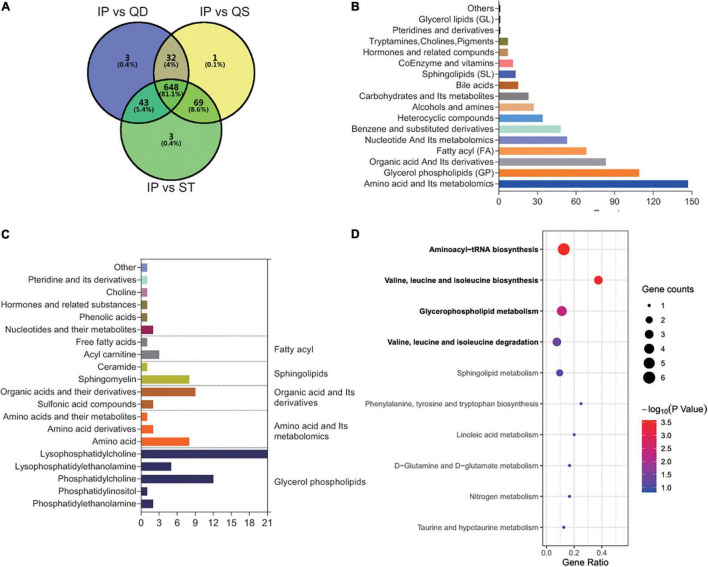
Comprehensive analysis to reveal the essence of IPBS. **(A)** The Venn diagram represents the relationship of comparison among IPBS and PST, PQD, and PQS. This diagram shows that most metabolites are non-differential between groups. **(B)** The column chart shows the main components of 648 non-differential regulated metabolites in the primary class. **(C)** The main secondary structure of the intersection between the top 100 metabolites in each group and 648 non-differential regulated metabolites. A total of 83 metabolites are included and glycerol phospholipids are the main components. **(D)** KEGG analysis of 83 metabolites. The gene ratio represents the enrichment factor, the bubble scale represents the number of different genes, the depth of the bubble color represents the *P*-value.

To reveal the essence of IPBS, non-differential metabolites between groups with VIP < 1 and fold change < 2 were selected for analysis. A Venn diagram was presented to describe the separate comparison between IPBS and its related concurrent syndromes. In total, 648 metabolites were common compounds in the three comparisons (Figure 2A). These common metabolites mainly included amino acid and its metabolomics, glycerol phospholipids, organic acid, and its derivatives, and fatty acyl (Figure 2B). Common compounds between non-differential metabolites and the intersection of the top 100 expressed metabolites were screened, and the details are presented in Figure 2C. Glycerol phospholipids were also main components. Overall, 83 metabolites were enriched in KEGG pathway analysis. The significantly enriched pathways were the same as the above KEGG enrichment. Valine, leucine, and isoleucine biosynthesis, glycerophospholipid metabolism, and valine, leucine, and isoleucine degradation were the main pathways (Figure 2D). Along with the above analysis, the results revealed that disordered in valine, leucine, and isoleucine metabolism and glycerophospholipid metabolism were determinants of IPBS. In detail, the key metabolites were phosphatidylethanolamine, phosphatidylcholine, 1-acyl-sn-glycero-3-phosphocholine, L-leucine, L-isoleucine, and L-valine.

### Comparison Between Intermingled Phlegm and Blood Stasis and Phlegm Stasis and Toxin

**FIGURE 3:**
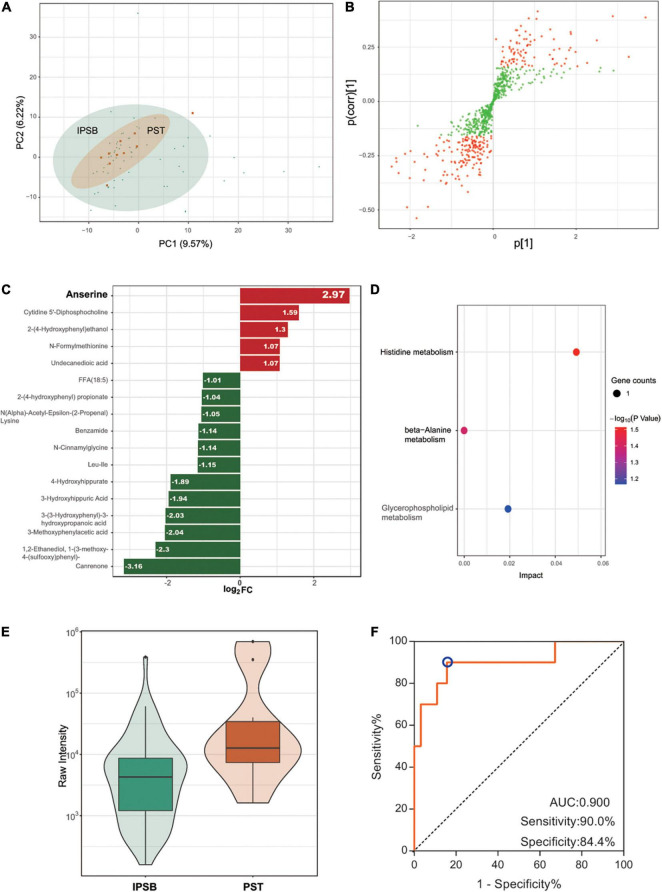
Comparison between IPBS and PST. **(A)** The PCA plot between IPBS and PST. Each point in the graph represents a sample, the different colors of the points represent the grouping of the samples, the differences are not distinct in the plot. **(B)** OPLS-SPlot between IPBS and PST. VIP > 1, red plots represent different regulated metabolites. **(C)** The column chart shows different regulated metabolites between IPBS and PST. Red bars represent upregulated metabolites in PST, green bars represent downregulated metabolites in PST. Data are shown in log2 scale. **(D)** KEGG analysis of different regulated metabolites between IPBS and PST; histidine metabolism, beta-alanine metabolism, and glycerophospholipid metabolism were the main enriched pathways. **(E)** The violin diagram shows the most significantly upregulated metabolites. Anserine is the top upregulated metabolite in PST. **(F)** ROC curve of the prediction model to identify IPBS and PST. AUC is 0.9, sensitivity is 0.9, and specificity is 0.844; the blue circle represents the truncation value.

For the relationship between main symptoms and coexisting symptoms, common content accounted for a large proportion. Thus, differences were not distinct in the PCA plot (Figure 3A). However, we also detected differently regulated metabolites in OPLS-Slot with VIP > 1 between the two groups of samples (Figure 3B). A total of 17 different metabolites existed between the two groups with VIP > 1 and fold change > 2. The most significant differently expressed compounds were anserine and canrenone (Figure 3C). KEGG pathway enrichment analysis of these different metabolites revealed that histidine metabolism, beta-alanine metabolism, and glycerophospholipid metabolism were the main enriched pathways (Figure 3D). Anserine was the only common metabolite in the three pathways. Its expression was higher in PST (Figure 3E). To accurate identify the difference between IPBS and PST, a prediction model with an AUC of 0.9, sensitivity of 0.9, and specificity of 0.844 was obtained (Figure 3F). The model was constructed by 0.00000028 × 3-(3-Hydroxyphenyl)-3-hydroxypropanoic acid -0.000003 × 1,2-Ethanediol,1-(3-methoxy-4-(sulfooxy) phenyl) + 0.000007 × anserine – 0.000056 × FFA (18:5) – 0.000041 × benzamide.

### Comparison Between Intermingled Phlegm and Blood Stasis and Phlegm Stasis and Qi Stagnation

**FIGURE 4:**
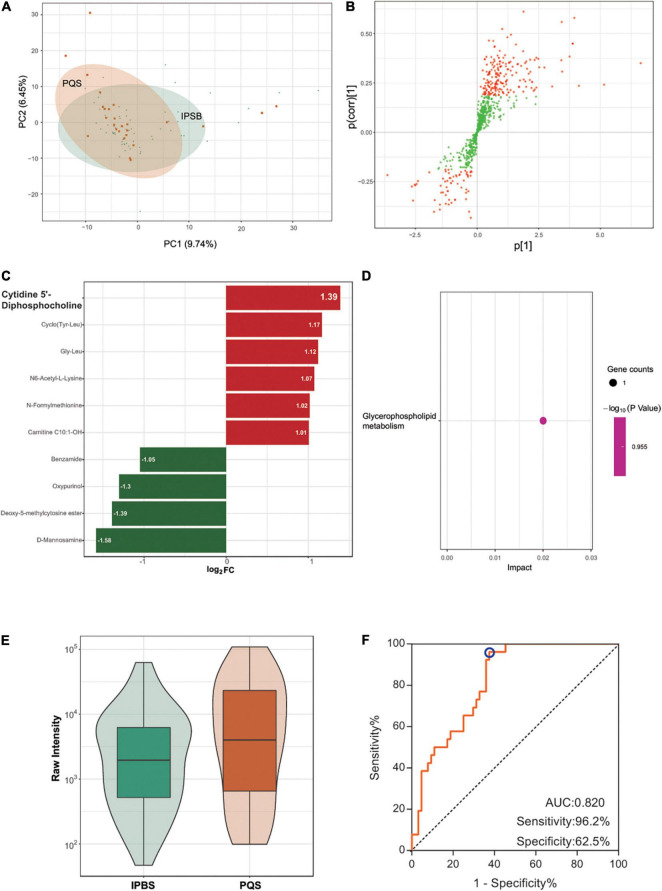
Comparison between IPBS and PQS. **(A)** The PCA plot between IPBS and PQS. Each point in the graph represents a sample, the different colors of the points represent the grouping of the samples, the differences are not distinct in the plot. **(B)** OPLS-SPlot between IPBS and PST. VIP > 1, red plots represent different regulated metabolites. **(C)** The column chart shows different regulated metabolites between IPBS and PST. Red bars represent upregulated metabolites in PQS, green bars represent downregulated metabolites in PQS. Data are shown in log2 scale. **(D)** KEGG analysis of different regulated metabolites between IPBS and PQS; glycerophospholipid metabolism was the main enriched pathway. **(E)** The violin diagram shows the most significantly upregulated metabolites. Cytidine 5′-diphosphocholine is the top upregulated metabolite in PQS. **(F)** ROC curve of the prediction model to identify IPBS and PQS. AUC is 0.82, sensitivity is 0.962, and specificity is 0.625; the blue circle represents the truncation value.

The PCA plot of the comparison was partially distinct (Figure 4A), which meant a relative difference between IPBS and PQS. Differently regulated metabolites between the two compared groups of samples were presented in the OPLS-SPlot with VIP > 1 (Figure 4B). Ten different metabolites existed between the two groups with VIP > 1 and fold change > 2. The most significant differently expressed compounds were cytidine 5′-diphosphocholine and D-mannosamine (Figure 4C). KEGG analysis revealed that glycerophospholipid metabolism was the main enriched pathway (Figure 4D). Cytidine 5′-diphosphocholine was the only metabolite in the pathway. Its expression was higher in PQS (Figure 4E). To identify the difference between IPBS and PQS, an accurate identification model was obtained with an AUC of 0.82, sensitivity of 0.962, and specificity of 0.625 (Figure 4F). The model was constructed by -0.000003 × N6-acetyl-L-lysine + 0.000004 × Gly Leu–0.000032 × benzamide + 0.000017 × cytidine 5′-diphosphocholine.

### Comparison Between Intermingled Phlegm and Blood Stasis and Phlegm Stasis and Qi Deficiency

**FIGURE 5:**
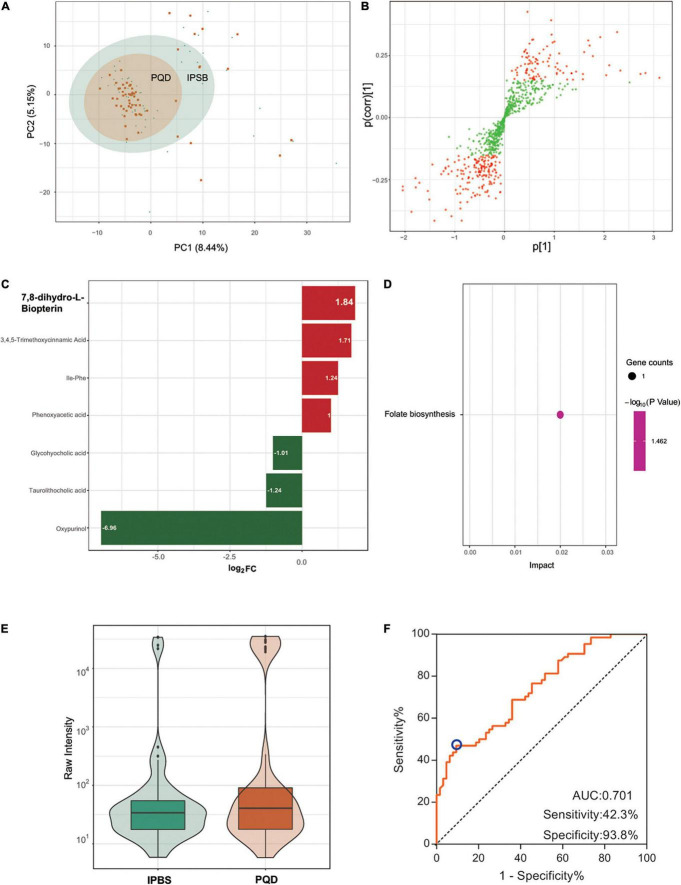
Comparison between IPBS and PQD. **(A)** The PCA plot between IPBS and PQD. Each point in the graph represents a sample, the different colors of the points represent the grouping of the samples, the differences are not distinct in the plot. **(B)** OPLS-SPlot between IPBS and PQD. VIP > 1, red plots represent different regulated metabolites. **(C)** The column chart shows different regulated metabolites between IPBS and PQD. Red bars represent upregulated metabolites in PQD, green bars represent downregulated metabolites in PQD. Data are shown in log2 scale. **(D)** KEGG analysis of different regulated metabolites between IPBS and PQD; folate biosynthesis was the most enriched pathway. **(E)** The violin diagram shows the most significantly upregulated metabolites. 7,8-dihydro-L-biopterin is the top upregulated metabolite in PQD. **(F)** ROC curve of the prediction model to identify IPBS and PQD. AUC is 0.701, sensitivity is 0.423, and specificity is 0.938; the blue circle represents the truncation value.

The PCA plot of the comparison was not distinct (Figure 5A), which meant there was a difficulty in separating IPBS from PQD. Differently regulated metabolites between the two compared groups of samples were presented in the OPLS-SPlot with VIP > 1 (Figure 5B). Combined with fold change > 2, only six different metabolites were detected in the comparison. The most significant differently regulated compounds were 7,8-dihydro-L-biopterin and oxypurinol (Figure 5C). KEGG analysis revealed that folate biosynthesis was the main enriched pathway (Figure 5D). 7,8-dihydro-L-biopterin was the only metabolite in the pathway and it was more expressed in IPBS (Figure 5E). It was difficult to identify the difference between IPBS and PQD. And an identification model with low accuracy was obtained, the AUC was 0.701, sensitivity was 0.423, and specificity was 0.938 (Figure 5F). The model was constructed by 0.0000006 × Ile-Phe + 0.000044 × glycohyocholic acid–0.000005 × oxypurinol – 0.0000007 × phenoxyacetic acid + 0.000117 × 3,4,5-trimethoxycinnamic–0.000014 × acid 7,8-dihydro-L-biopterin.

## Discussion

Previous research reported that metabonomics was a valuable method in elaborating the mechanism of coronary heart disease or TCM syndromes (12). Shi et al. detected 39 endogenous metabolites between unstable angina pectoris patients and healthy people, they conjectured that dysregulated metabolic processes of amino acids, glucose, or lipids might be involved in the pathogenesis of unstable angina pectoris (13). Cai et al. reported the function of a traditional Chinese medicine Tongmai Yangxin Pill (TMYX) in treatment of angina and arrhythmia through metabolomics analysis. They found 10 biomarkers were reversed to the normal after TMYX treatment, these metabolites were involved in energy metabolism, alanine, aspartate, and glutamate metabolism, oxidative stress, and inflammation (14). Guo et al. detected 17 potential biomarkers associating with altered Chinese medicine syndromes with ultrafiltration. Sphingolipid metabolism and phospholipid metabolism were the main enriched pathways (15). However, there was no detailed study about IPBS and its concurrent syndromes. Our study firstly analyzed the metabolic characteristics of IPBS and its concurrent syndromes in stable angina pectoris of coronary heart disease. We used metabolites to reveal inside changes in patients that reflect the external appearance of TCM syndromes.

We found that glycerol phospholipid metabolism was the key pathway of IPBS, which was consistent with the above reports. In this pathway, phosphoglyceride was the major component, it could be hydrolyzed by phospholipases PLA1 or PLA2 to produce lysophospholipid (LP), which functions in destroying the cell membrane. Lysophospholipids mainly include lysophosphatidylcholine, lysophosphatidic acid, and some sheath phospholipids. Studies showed that these lysophospholipids activated the PPAR γ pathway involved in atherosclerosis (16). Because atherosclerosis was the basis of angina pectoris, we inferred that glycerol phospholipid metabolism could be used to represent the basis of IPBS and its concurrent syndromes. This key enriched metabolites in glycerol phospholipid metabolism included phosphotidylethanolamine, phosphotidylcholine, and 1-acyl-sn-glycoro-3-phosphocholine, which will be of great significance in heart disease progression and monitoring.

Besides, we also found valine, leucine, and isoleucine metabolism was a main enriched pathway, and the branched chain amino acids (BBCAs) L-leucine, L-isoleucine, and L-valine were major different metabolites. A report showed that BCAAs provided nitrogen to glutamine and alanine synthesis for muscles, and their consumption increased in heart diseases. In myocardial ischemia, BCAAs were derived from the mobilization of muscle protein and were important alternative energy substrates for the synthesis and metabolism of myocardial protein (17). A study of amino acid metabolomics revealed that contents of valine, histidine, ornithine, and other amino acids were raised in the coronary heart disease group with Qi stagnation and blood stasis syndrome or Qi deficiency and blood stasis syndrome (18). Another study reported that the level of BCAAs increased with the aggravation of ischemia of the heart, and leucine was the most obvious increased amino acid, with the potential to be a clinical reference index for the occurrence and prognosis of ischemic heart disease (19). Besides, leucine was reported to induce cardioprotection *in vitro* by promoting mitochondrial function *via* mTOR and Opa-1 signaling (20). This research indicated that a disorder in valine, leucine, and isoleucine metabolism regulated by L-leucine, L-isoleucine, and L-valine could reflect the essence of stable angina.

There were some limitations in the study. A normal population as the control was not set, because of the difficulty in the identification of TCM symptoms and confounding factors in the normal population. Thus, we used the top 100 representative expressed metabolites and non-differential metabolites among the four tested groups to reflect the essence of IPBS and to distinguish main and concurrent syndromes. Then we focused on glycerol phospholipid metabolism, valine, leucine, and isoleucine metabolism, and their key metabolites, which prepared the basis for the follow-up drug action mechanism. For the main and concurrent relationship, it was difficult to distinguish IPBS with PQD, PQS, and PST. Besides, the ability of a single metabolite to distinguish main and concurrent symptoms was low. Therefore, we used the combination of multiple metabolites to draw an ROC curve, which was conducive to obtaining a high distinguishing ability. The ROC curve could display the distinction between IPBS with PST, but not for PQD, which needed to be distinguished with the help of appearance and other tools.

In this study, we found IPBS may result from a disorder of glycerol and phospholipid metabolism and valine, leucine, and isoleucine metabolism. And the involved phosphodylethanolamine, phosphodylcholine, 1-acyl-sn-glycoro-3-phosphocholine, L-leucine, L-isoleucine, and L-valine had the potential to be biomarkers in clinical auxiliary diagnosis for IPBS. Although, there are still some problems in the collection of clinical serum, such as sample preservation, the acceptance of serology among TCM patients, as well as the cost of metabolic testing. With the improvement of technology and the continuous progression of testing methods, and implementation of large-scale crowd validation in confirming the diagnostic efficacy of these metabolites, we expect detection of these metabolites to make up for the subjectivity of diagnosis and distinguish concurrent syndromes.

## Conclusion

Metabonomics can reflect the characteristics of IPBS and its concurrent syndromes in stable angina pectoris of coronary heart disease. IPBS may be related to the disorder of glycerol and phospholipid metabolism and valine, leucine, and isoleucine metabolism regulated by phosphodylethanolamine, phosphodylcholine, 1-acyl-sn-glycoro-3-phosphocholine, L-leucine, L-isoleucine, and L-valine. Differential metabolites easily distinguished IPBS with PBT, but not with PQD.

## Data Availability Statement

The datasets presented in this study can be found in online repositories. The names of the repository/repositories and accession number(s) can be found in the article/supplementary material.

## Ethics Statement

The studies involving human participants were reviewed and approved by the Ethics Committee of 12 Research Centers (Affiliated Hospital of Liaoning University of Traditional Chinese Medicine, Dongzhimen Hospital of Beijing University of Traditional Chinese Medicine, Affiliated Hospital of Shaanxi University of Traditional Chinese Medicine, the First Affiliated Hospital of Guangxi University of Traditional Chinese Medicine, Yunnan Hospital of Traditional Chinese Medicine, Affiliated Hospital of Southwest Medical University, Longhua Hospital Affiliated to Shanghai University of Traditional Chinese Medicine, the First Affiliated Hospital of Guangzhou University of Traditional Chinese Medicine, Hubei Hospital of Traditional Chinese Medicine, Gansu Central Hospital, the Affiliated Hospital of Chengdu University of traditional Chinese Medicine and the First Affiliated Hospital of Heilongjiang University of traditional Chinese Medicine). Approved and supervised the research protocol. The patients/participants provided their written informed consent to participate in this study.

## Author Contributions

ZM designed the experiments and provided the article idea. LZh wrote the manuscript and provided part of the article idea. ZY designed the experiments and edited the manuscript. YY, ZY, AY, and LB provided clinical input and completed data management and analysis. All authors contributed to manuscript revision, and read and approved the submitted version.

## Conflict of Interest

The authors declare that the research was conducted in the absence of any commercial or financial relationships that could be construed as a potential conflict of interest.

## Publisher’s Note

All claims expressed in this article are solely those of the authors and do not necessarily represent those of their affiliated organizations, or those of the publisher, the editors and the reviewers. Any product that may be evaluated in this article, or claim that may be made by its manufacturer, is not guaranteed or endorsed by the publisher.

## Footnotes

1
https://sciex.com.cn/

2
https://sciex.com/

3
www.ebi.ac.uk/metabolights/MTBLS4626

4
http://www.r-project.org/

5
http://www.kegg.jp/kegg/compound/

6
https://www.metaboanalyst.ca/MetaboAnalyst/
